# Sacral nerve modulation for patients with fecal incontinence: long-term outcome and effects on sexual function

**DOI:** 10.1007/s13304-023-01570-z

**Published:** 2023-07-13

**Authors:** Luigi Brusciano, Antonio Brillantino, Gianluca Pellino, Franco Marinello, Coen IM Baeten, Alex Digesu, Gabriele Naldini, Claudio Gambardella, Francesco Saverio Lucido, Alessandro Sturiale, Giorgia Gualtieri, Stefan Riss, Ludovico Docimo

**Affiliations:** 1 Department of Advanced Medical and Surgical Sciences, University of Campania Luigi Vanvitelli, Naples, Italy; 2 III Department of Surgery, “A. Cardarelli” Hospital, Naples, Italy; 3 Colorectal Surgery, Vall d’Hebron University Hospital, Universitat Autònoma de Barcelona, Barcelona, Spain; 4 Department of Surgery, Groene Hart Hospital, Gouda, The Netherlands; 5 Department of Urogynaecology, Imperial College NHS Healthcare, London, UK; 6 Proctology and Perineal Surgical Unit - Proctology and Pelvic Floor Multidisciplinary Clinical Center, Universitary Hospital of Pisa, Pisa, Italy; 7 Department of Surgery, Medical University Vienna, Vienna, Austria

**Keywords:** Sacral nerve modulation, Fecal incontinence, Urinary incontinence, Double incontinence, Urgency

## Abstract

Sacral nerve modulation has become an established treatment for fecal and urinary incontinence, and sexual disorders. The objective of this study was to evaluate the long-term outcome of sacral neuromodulation in patients with fecal or combined fecal and urinary incontinence (double incontinence), assessing its safety, efficacy, and impact on quality of life and sexual function. This was a multicentric, retrospective, cohort study including patients with fecal or double incontinence who received sacral neuromodulation at seven European centers between 2007 and 2017 and completed a 5-year follow-up. The main outcome measures included improvements of incontinence symptoms and quality of life compared with baseline, evaluated using validated tools and questionnaires at 1-, 6-, 12-, 36- and 60-month follow-up. 108 (102 women, mean age 62.4 ± 13.4 years) patients were recruited, of whom 88 (81.4%) underwent definitive implantation of the pacemaker. Patients’ baseline median Cleveland Clinic Incontinence Score was 15 (10–18); it decreased to 2 (1–4) and 1 (1–2) at the 12- and 36-month follow-up (*p* < 0.0001), remaining stable at the 5-year follow-up. Fecal incontinence quality of life score improved significantly. All patients with sexual dysfunction (*n* = 48) at baseline reported symptom resolution at the 5-year follow-up. The study was limited by the retrospective design and the relatively small patient sample. Sacral nerve modulation is an effective treatment for fecal and double incontinence, achieving satisfactory long-term success rates, with resolution of concomitant sexual dysfunction.

## Introduction

Fecal incontinence (FI) is a humiliating and devastating condition, which deeply impacts quality of life to an extent difficult to quantify, as it is often hidden and results in social stigma [1–3]. Any treatment attempt for FI, even when not completely satisfactory, can be justified if able to improve the patient’s quality of life [4, 5]. Among the possible surgical approaches, repairs of any anal sphincter defect are considered effective only in the short term, whereas anal sphincter replacement procedures, which might prove effective in the long term, are usually burdened by a high rate of adverse events or re-operations [6–8] .

Medical treatment and rehabilitation are strictly indicated only in FI associated with functional–physiatric impairments, and show disappointing results when used as an isolated rather than a combined or integrative treatment [9, 10].

Sacral nerve modulation (SNM) has become an established treatment for several functional disorders of the pelvic organs such as FI, urinary incontinence (UI) and sexual dysfunction, acting by neuromodulating the nerve roots pertaining to the involved nervous districts [11, 12]. However, data on long-term results of SNS are still scarce, and its impact on sexual function needs to be elucidated.

The aim of this study was to evaluate the long-term outcome of SNM in the treatment of patients with FI, either alone or combined with UI, assessing its safety, efficacy, and impact on quality of life and sexual function.

## Methods

### Patient enrollment and design of the study

The research consisted of an international, multicenter, retrospective cohort study including data on patients with FI observed between 2007 and 2017 at seven European centers (University of Campania “Luigi Vanvitelli”, Naples, Italy, “A. Cardarelli” Hospital, Naples, Italy, Hospital Universitari “Vall d’Hebrón” Universitat Autònoma de Barcelona, Barcelona, Spain, Groene Hart Hospital, Gouda, The Netherlands, Imperial College NHS Healthcare, London, UK, Universitary Hospital of Pisa, Pisa, Italy, Medical University Vienna, Vienna, Austria) who underwent treatment with SNM. All the participating centers were required to have at least 5-year follow-up data available.

The study is reported according to the STrengthening the Reporting of OBservational studies in Epidemiology (STROBE) statement for cohort studies [13] and was conducted according to the Declaration of Helsinki. All the patients provided written consent for surgical intervention.

### Inclusion and exclusion criteria

Inclusion criteria were: patients over 18 years of age, affected by clinical FI (defined as at least one incontinence episode per week reported for a period of time longer than 6 months) or affected by both FI and UI, who had received re-education and physiatrist consultation (see below).

Patients with any condition potentially responsible of temporary FI (active anal fistula, recent rectal resection or anal sphincterotomy, recent proctologic surgery), or patients with a complete rectal prolapse, inflammatory bowel disease, diarrhea refractory to drugs or diet, or who had had vaginal or cesarean delivery in the previous 12 months were excluded from the study.

### Clinical assessment

All candidates to SNM underwent a baseline physical examination by an expert coloproctologist. A clinical assessment consisting of a bowel habit diary with severity, onset, duration, and clinical subtype of FI (passive or urge incontinence) was routinely performed at all centers. Stool consistency was evaluated at first examination according to the Bristol scale [14]. Severity of FI was assessed with the Cleveland Clinic Incontinence Score system (CCIS) [15, 16], and its impact on quality of life was evaluated with the Fecal Incontinence Quality of Life (FIQoL) [17]. Patients affected by urge incontinence were asked about the time to postponing defecation, counting the seconds from the defecation stimulus onset to the actual defecation time or incontinence episode following the stimulus.

Patients were asked about sexual and urinary function. In case of symptoms attributable to UI, they were further evaluated with the International Consultation on Incontinence Questionnaire‐Urinary Incontinence Short Form (ICIQ-UI-SF) [18]. In detail, values greater than one were considered diagnostic for urinary incontinence, identifying patients with double incontinence (DI), defined as a combination of FI and UI.

All patients underwent an instrumental evaluation with recto-sigmoidoscopy, anorectal manometry, and three-dimensional endoanal ultrasound [19–22] and a physiatrist assessment. In addition, all the patients with additional symptoms attributable to obstructed defecation syndrome (ODS) underwent defecography.

### Re-education phase and physiatry assessment

As first-line treatment, all patients underwent a re-education phase consisting in diet counseling, measures to augment the pelvic floor self-perception, and to provide the right position or breathing dynamics during defecation, with the aim of correcting the first steps of a physiologic defecation and reaching a complete and satisfactory emptying of the rectum.

Along with the re-education phase, patients were evaluated according to the physiatric parameters indicating thorax–abdominal–sphincter harmonization [15]. If any of those parameters were abnormal, patients were addressed to pelvic floor rehabilitation. All patients with persisting FI or DI following the re-education phase and/or rehabilitation were offered peripheral nerve evaluation (PNE) test.

### Operative procedure

A consistent approach is used for PNE and SNM at all centers and consisted of the following steps.

Patients are positioned in the prone position with buttocks taped apart so that the anus can be observed during electro stimulation. After local anesthesia infiltration, the needle is placed in correspondence of the S3 or S4 foramen. With the needle in place, a lateral radiographic scanning of the sacrum is performed to confirm the needle position and allow further adjustments where appropriate. Testing stimulation is then performed with the external pulse generator, to achieve an anal motor response or toe/forefoot response at a low current (< 2 mA). The needle stylet is removed to insert the directional guidewire, the electrode is placed, and its final location is checked with fluoroscopy. Once the tined lead is positioned, the electrode is tunneled to a pocket in the contralateral buttock, together with the percutaneous extension used for the external stimulation during the test period. The patients are given a remote to control the amplitude of the device stimulus.

After 1 month from the PNE test, in case of reported improvement of symptoms as confirmed by the decrease in CCIS, the definitive pacemaker is implanted (Interstim, code 3023, or Interstim II, code 3058 devices, Medtronic, Dublin, Ireland). A subcutaneous pocket is created to contain the device, making sure it is large enough to fit it comfortably. The electrode is then connected to an impulse generator [19].

### Follow-up and outcome measures

For the purpose of this study, only patients who received SNM for at least 5 years were considered. The presence, severity, and frequency of FI episodes were evaluated during outpatient visits by CCIS score 1 month after the PNE test, and 1 and 6 months after pacemaker implantation. Subsequent evaluations were performed after 1 year, and two times yearly thereafter, and they were conducted by emailing patients dedicated forms to be returned within 6 days. This follow-up timing was also used for the quality-of-life assessment with FIQoL. Patients affected by DI were further evaluated with ICIQ-UI-SF questionnaire at the same follow-up intervals.

The manometric evaluation was repeated in all the patients 6 months after pacemaker implantation.

The primary outcome of the study consisted in improvement of FI or DI symptoms and quality of life over time and at the 5-year follow-up; outcome measures consisted of CCIS score, ICIQ-UI-SF, and FIQoL.

The secondary outcome was the evaluation of post-procedural sexual function in those patients who reported sexual dysfunction during preoperative examination.

### Statistical analysis

Statistical analysis was performed using Excel 2011® (Microsoft, Redmont, WA). Categorical data are reported as absolute numbers with percentages. Continuous data are reported as medians with ranges or means ± standard deviation (SD), according to data distribution. The differences between paired results were analyzed by the Wilcoxon matched pairs test or by the paired *t* test, when indicated. *P* values < 0.05 were considered statistically significant.

## Results

### Study population

**Fig. 1:**
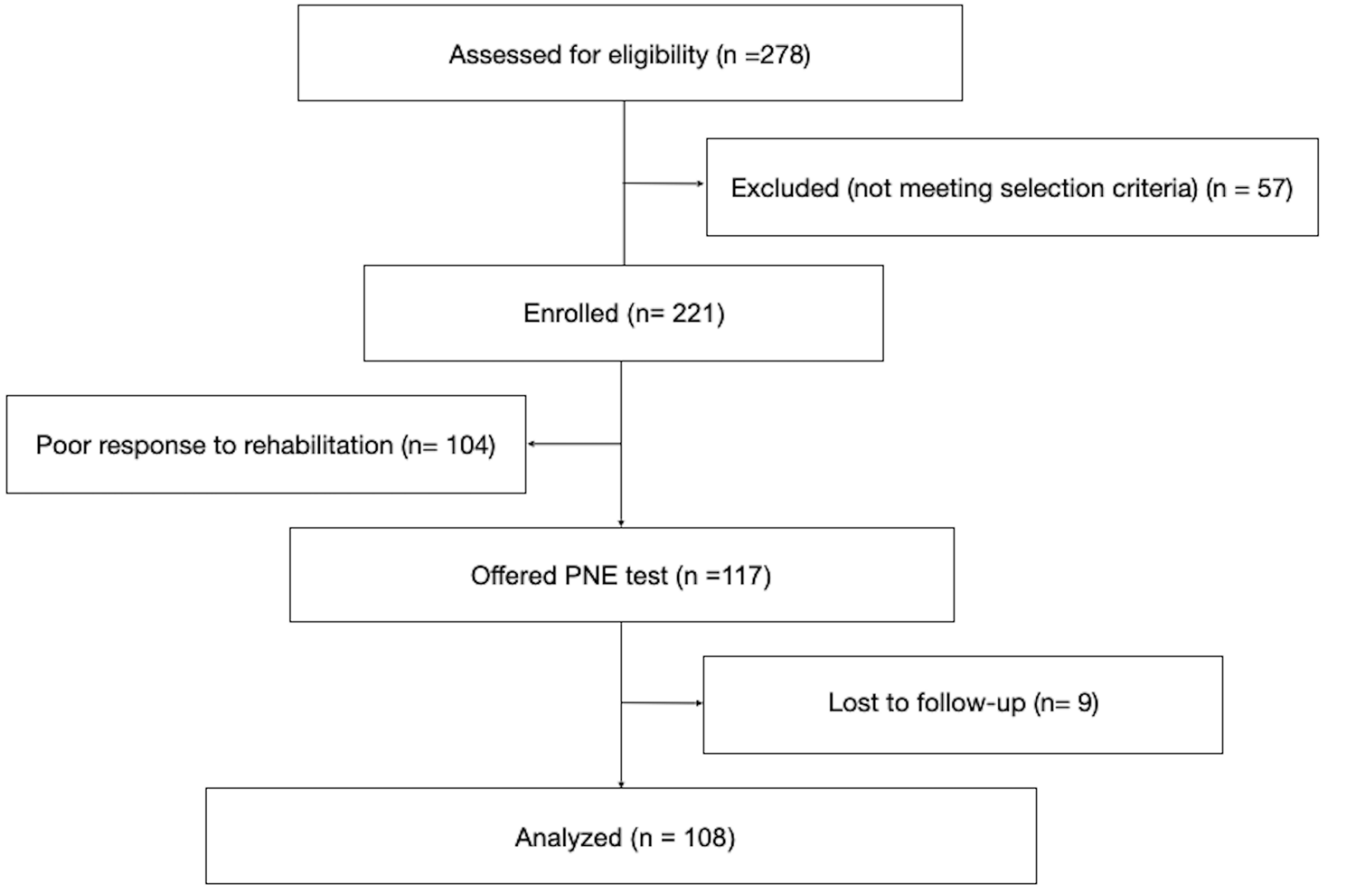
Flowchart of patient inclusion in the study. PNE: peripheral nerve evaluation

Out of 278 patients assessed for eligibility, 221 (79.4%) met the inclusion criteria and were enrolled in the study. Of these, 117 (53%) patients with persisting FI or DI following the re-education phase and/or rehabilitation were offered PNE test. Out of them, nil required removal of the pacemaker, but nine (7.7%) were lost to follow-up. Hence, 108 (92.3%, 102 women, 6 men, mean age 62.4 ± 13.4 years) patients completed the 5-year follow-up and were included in the analysis (Fig. 1).

### Baseline features

Among the analyzed patients, the most frequent registered comorbidity was hypertension (44.4%), followed by diabetes mellitus (35.1%), other cardiovascular diseases (19.4%), chronic obstructive pulmonary diseases (18.5%), autoimmunity (17.5%), renal failure (11.1%), and neurologic diseases (11.1%). Of 102 women, 62 (60.8%) were sexually active.

No patient showed signs of proctitis at endoscopy.

Twelve (11.1%) female patients with associated ODS symptoms underwent defecography, showing in all the cases signs of rectal intussusception combined with rectocele in 83.3% (*n* = 10) of them. All the patients with fecal incontinence associated with ODS symptoms showed endosonographic signs of anal sphincter lesions.

The main manometric and endosonographic characteristics of included patients are shown in Table 1.

**Table 1:** Main manometric and endosonographic features of the analyzed patients

Features	
No sphincter injuries, *n*	28 (25.9)
External sphincter lesions, *n*	80 (74%)
External and internal sphincter lesions, *n*	28 (25.9)
Anal resting pressure, mmHg	52 (40–65)
Maximum voluntary contraction, mm	95 (80–100)
Maximum voluntary contraction duration, s	10 (8–12)
Rectal sensitivity, cc - First threshold - Second threshold - Third threshold	25 (20–30) 50 (40–60) 90 (70–100)

Values are given as absolute numbers (%) or medians (range)

At endoanal ultrasound, 28 (26%) examinations were negative for sphincter injuries, whereas 80 (74%) patients had external sphincter lesions. In detail, 45 (41.6%) patients presented external sphincter lesions with transversal extension ≤ 45° and mean longitudinal extension of 15 ± 2.17 mm; 23 (21.2%) patients had external sphincter lesions with transversal extension between 45° and 70° and mean length of 16 ± 3.09 mm; 12 (111.1%) patients had injuries to the external sphincter with transversal extension between 82° and 105° and mean longitudinal extension of 16 ± 4.4 mm. Out of 80 patients with external sphincter damage, 28 (35%) had associated internal sphincter lesions.

Baseline anorectal manometry recorded a median resting pressure of 52 (40–65) mmHg and a median maximum voluntary contraction of 95 (80–100) mmHg, with a median duration of 10 (8–12) s. In terms of rectal sensibility, the median first threshold was 25 (20–30) cc, the second was 50 (40–60) cc, and the third was 90 (70–100) cc.

Overall, fecal incontinence was ascribable to anal sphincter lesions in 74% (*n* = 80) of cases, whereas it was considered secondary to neuropathy or autoimmunity in 14.9% (*n* = 16) and idiopathic in 11.1% (*n* = 12) of the cases. The anal sphincter lesions were attributable to previous obstetrical trauma in 20 (25%) cases.

### Surgery

Among the 108 patients who underwent testing, 88 were implanted a definitive pacemaker (61 of these were diagnosed with FI and 27 with DI) resulting in a total SNM screening success rate of 81.4%. Of the 108 patients screened, 86 (80%) had the electrode implanted at the S3 root (80% on the left and 20% on the right side) and 22 (20%) patients on the S4 root.

**Fig. 2:**
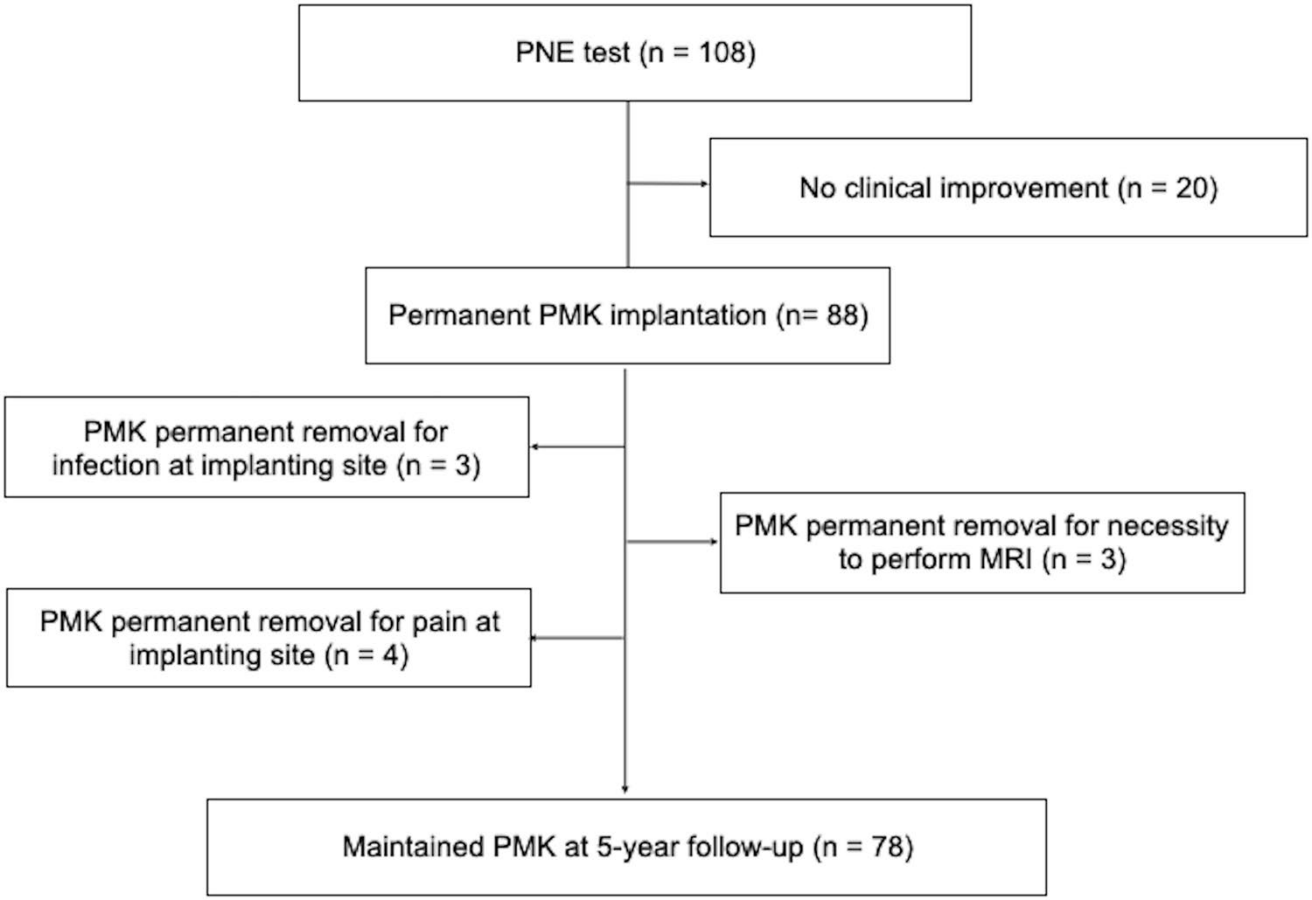
Evolution of patients and reasons to remove the pacemaker. PMK: pacemaker; PNE: peripheral nerve evaluation

Among the 88 patients permanently implanted, the SNS was removed in three (3.4%) for infection at the implanting site and in three (3.4%) for the need of undergoing pelvic MRI. All these patients maintained anal continence after pacemaker removal. In nine (9%) patients with permanently implanted SNM, the pacemaker was removed and re-implanted because of pain at the implanting site, and in four of these patients the pacemaker was permanently removed because pain was not resolved after re-implantation. Of these patients, two (50%) maintained anal continence after removal of the pacemaker. Overall, 78 (72%) patients maintained the pacemaker implantation at 5 years follow-up. A pacemaker implantation maintaining flowchart is reported in Fig. 2.

### Postoperative manometric results

Median resting pressure increased to 66 (55–75) mmHg 6 months after implantation (*p* < 0.0001). Median maximum voluntary contraction increased to 110 (90–122) mmHg after 6 months (*p* = 0.0006). At the same follow-up interval, its duration increased from 10 (8–12) s to 16 (14–18) s (*p* < 0.0001). Rectal sensibility increased as follows: median first threshold was 35 (24–40) cc after 6 months from PMK implantation (*p* = 0.0067), median second threshold increased to 65 (57–73) cc (*p* = 0.0001), and median third threshold to 140 (135–155) cc (*p* = 0.0001) (Table 2).

**Table 2:** Postoperative vs preoperative manometric results

Features	Preoperative	Postoperative	*P*
Anal resting pressure, mmHg	52 (40–65)	66 (55–75)	< 0.001
Maximum voluntary contraction, mm	95 (80–100)	110 (90–122)	< 0.001
Maximum voluntary contraction duration, s	10 (8–12)	16 (14–18)	< 0.001
Rectal sensitivity, cc - First threshold - Second threshold - Third threshold	25 cc (20–30) 50 cc (40–60) 90 cc (70–100)	35 (24–40) 65 (57–73) 149 (135–155)	< 0.001

Values are given as medians (range); Wilcoxon matched pairs test

### Primary aim: fecal incontinence and quality of life

**Fig. 3:**
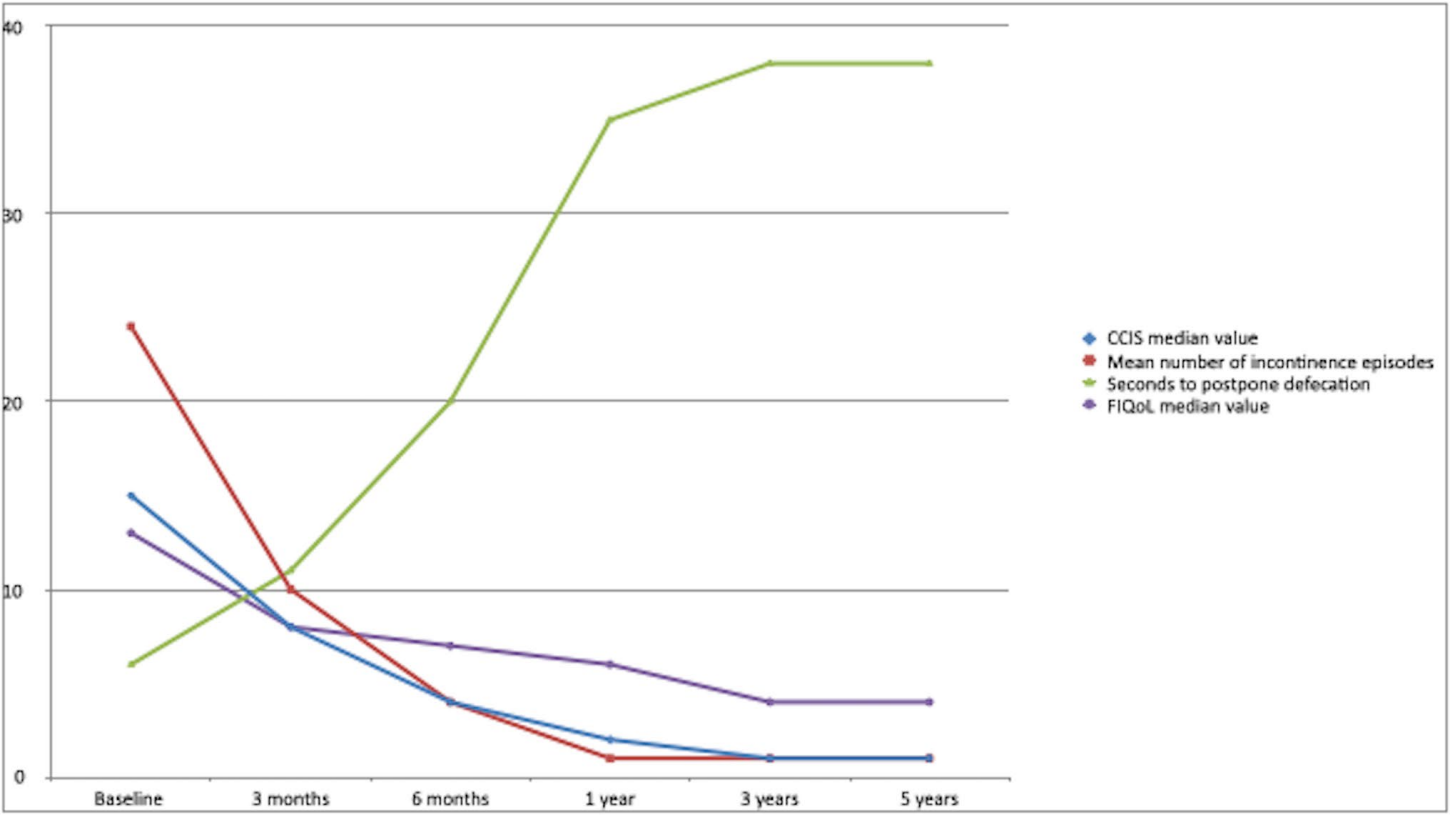
Trends of symptoms over time after pacemaker implantation. CCIS: Cleveland Clinic Incontinence Scale

Figure 3 shows the main outcomes after pacemaker implantation. Patient’s baseline CCIS median value was 15 (10–18) and decreased at 8 (4–12), 4 (1–8), 2 (1–4) and 1 (1–2), respectively, after 1, 6, 12 and 36 months (*p* < 0.0001), maintaining the reached value at the 5-year follow-up. The mean number of incontinence episodes per 4 weeks decreased from 24 ± 3.6 at baseline to 10 ± 3 after the first month of follow-up in patients who were permanently implanted with the PMK (*p* < 0.0001). This value decreased to 4 ± 2.5 per month at 6 months follow-up, and 1 ± 0.5 per month at the 1-, 3-, and 5-year follow-up. The median time patients could postpone defecation from the onset of sensation of urge increased from 6 (3–7) s at baseline to 11 (9–13) s after the SNS screening period (one month) (*p* = 0.001). In all patients, the time to postpone defecation after being implanted at the 6-, 12-, and 36-month follow-up was higher than baseline, with median values of 20 (18–22) s, 35 (32–37) s, and 38 (36–39) s, maintaining the same values at the 5-year follow-up (p < 0.0001).

All four domains’ mean values of the FIQoL decreased significantly, meaning that the quality of life increased. Compared from baseline to 12 months follow-up, lifestyle decreased from 3.6 ± 1.5 to 2.1 ± 0.5, coping from 3 ± 1.4 to 1.5 ± 0.6, embarrassment from 3 ± 2 to 1.5 ± 3.6, and depression from 3.6 ± 1.8 to 2.7 ± 0.4. The corresponding mean values were 2 ± 0.5 (*p* < 0.001; paired test), 1.1 ± 0.6 (*p* < 0.001; paired test), 1 ± 0.3 (*p* < 0.001; paired test), and 2.3 ± 1.2 (*p* < 0.001; paired test) at 3 years and remained stable at the 5-year follow-up.

### Primary aim: urinary incontinence in patients with double incontinence

The median ICIQ-UI-SF value at baseline was 13 (9–15), which is defined as severe urinary incontinence, and decreased to 9 (6–14) 6 months after implantation, 6 (2–8) at the 1-year, and 5 (3–7) at 3-year follow-up (*p* < 0.0001), maintaining the same value at the 5-year follow-up. Urgency disappeared in 15 out of 31 women who originally reported it.

### Secondary aim: sexual function

Forty-eight female patients (44.4% of the screened patients) reported sexual dysfunction at the first visit. Thirty-eight (79.1%) had pain during penetration or intercourse, while 9 (18.7%) were afraid of incontinence during intercourse at baseline. All of the 48 patients were sexually active 1 year after implantation, and reported resolution of sexual dysfunction. The effects were maintained after 5 years.

## Discussion

The current multicentric study found satisfactory outcome of SNM in the treatment of both FI and DI, and sexual dysfunction. Results are maintained at long-term follow-up, and positively impact quality of life and social function of patients. Adverse events and need for re-operation and removal of the PMK occurred in a limited sample of patients, further demonstrating the safety of this procedure.

According to literature, although mild FI could be solved by dietary and behavioral modifications, a relevant proportion of patients affected by FI will need further treatment [23, 24].

Pelvic floor muscle rehabilitation proved to be useful [25], but the results are reported to be less encouraging in case of isolated internal anal sphincter weakness, overflow incontinence, highly impaired rectal sensation, or coexistent structural damage of the anal sphincters [26, 27]. In our series of patients poorly responsive to rehabilitation, SNS showed to be useful in decreasing the number of FI episodes, lowering the CCIS score, increasing the time to postpone defecation, and improving the FIQoL at the long-term follow-up.

According to previous studies [28–31], the SNM success rate in our series was considerable, reaching 76%, if patients undergoing pacemaker removal were considered. Our results are comparable to those previously reported by a European multicenter study, showing a long-term success rate of 71.3% [32]. Interestingly, the majority of patients experienced a complete response to the treatment within the first weeks and months after implantation, being able to report whether pacemaker was actually benefitting them or not.

In terms of adverse events, approximately 9% of patients underwent re-operation for the onset of local pain in the present study. This complication has been widely studied and similar figures have been reported in other studies [33–36], which suggested that it could be avoided by creating an optimal gluteal pocket during electrode implantation. In 9% of patients in this study, PMK was definitively removed for the onset of clinical conditions requiring spinal and pelvic MRI, for persisting pain or for infection at the implantation site. It is important to note that 80% of these patients maintained anal continence over time, suggesting that the anal continence could benefit from previous stimulation even after PMK removal. The reasons of this phenomenon are unclear; however, according to other authors, it could be speculated that a sufficiently long period of stimulation may induce cerebral neuroplasticity that restores neural circuitry to a pre-incontinence status and that may permit cessation of stimulation in selected cases [37]. Better understanding of the nerve activation patterns leading to the PMK efficacy could help to explain this persisting benefit after the PMK removal and to identify factors that could reduce the loss of efficacy of SNM over time.

Interestingly, in our series, a considerable percentage of patients showed rectal hypersensitivity associated with any type of incontinence and, considering that no patient showed endoscopic signs of proctitis, the reasons for this finding are unclear. However, according to current study results, sacral nerve modulation was able to improve all the median rectal sensory thresholds.

Lastly, in the current series, SNM was also effective in improving UI and sexual function. Patients treated with SNM for combined anal and urinary incontinence also reported a reduction of the ICIQ-UI-SF score, with resolution of urgency in almost 60% of them. Fear of incontinence during intercourse improved in all patients. Moreover, all the sexually active patients, who had had pain during intercourse at baseline, reported symptom resolution, suggesting a potential role of pacemaker implantation in the treatment of sexual function disorders.

### Study limitations and strengths

The current study, although multicentric, is limited by its retrospective design and by the relatively small sample size of the analyzed patients. Another limitation is represented by the employment of different manometric and endosonographic equipments that limited the comparability of the functional and morphologic findings. However, all patients were assessed, treated, and followed up with a rigorous methodology at referral centers with dedicated teams to treat FI, who collected data prospectively. This study offers novel information and perspectives on the safety and efficacy of SNM in the long term, as well as some factors that might lead to pacemaker removal; of note, this does not necessarily translate into worse outcomes in the long term. Also, most patients would experience immediate benefit after treatment. The fact that the achieved results are maintained in the long term even in some patients who required removal suggests an actual beneficial effect of neuromodulation. Resolution of any accompanying urinary or sexual disorder further adds to the expected benefits of SNM.

## Conclusions

SNM is a useful tool to treat FI and DI, achieving satisfactory long-term success rate in patients who did not benefit from conservative treatment and pelviperineal rehabilitation. Further studies are needed to better analyze and understand the pathophysiological mechanisms responsible for SNM effectiveness and to create protocols able to standardize indications to its use. The effects on SNM on sexual disorders are of great interest, especially in this population of patients, and merit further research.
